# How can biological databases support the new UN mechanism for benefit-sharing from digital sequence information?

**DOI:** 10.1038/s41597-026-07725-y

**Published:** 2026-06-24

**Authors:** Débora S. Raposo, Davide Faggionato, Barbara Ebert, Pablo Orozco, Guy Cochrane, Yiming Bao, Masanori Arita, Melania Muñoz-García, Jens Freitag, Tim Hirsch, Charles E. Cook, Rutger Vos, Lorenz Reimer, Sujeevan Ratnasingham, Mathieu Rouard, Sarah Brinkley, Matthias Lange, Uwe Scholz, Stephan Weise, Björn Usadel, Robert D. Finn, Chan-Ho Park, Aylin S. Haas, David Castle, Colman O’Cathail, Amber H. Scholz

**Affiliations:** 1 GFBio - German Federation for Biological Data e.V., Bremen, Germany; 2 Leibniz Institute DSMZ - German Collection of Microorganisms and Cell Cultures GmbH, Department of Science Policy & Internationalisation, Braunschweig, Germany; 3 European Molecular Biology Laboratory, European Bioinformatics Institute (EMBL-EBI), Hinxton, United Kingdom; 4 National Genomics Data Center, China National Center for Bioinformation and University of Chinese Academy of Sciences, Beijing, China; 5 Bioinformation and DDBJ Center, National Institute of Genetics, Mishima, Japan; 6 Leibniz Institute of Plant Genetics and Crop Plant Research (IPK), Gatersleben, Germany; 7 Tim Hirsch Consulting, London, United Kingdom; 8 Global Biodata Coalition, Hinxton, United Kingdom; 9 Data Competence Center, Naturalis Biodiversity Center, Leiden, Netherlands; 10 University of Guelph, Guelph, Canada; 11 Bioversity International, Montpellier, France; 12 The Periodic Table of Food Initiative, Bioversity International, Rome, Italy; 13 Forschungszentrum Jülich, Jülich, Germany; 14 Fisheries Science Institute, Chonnam National University, Yeosu, South Korea; 15 School of Public Administration, University of Victoria, Victoria, Canada

**Keywords:** Policy, Research data

## Abstract

In October 2024, Parties to the United Nations Convention on Biological Diversity agreed to a new multilateral mechanism to fund biodiversity conservation through the sharing of benefits from open biodiversity data. Biological databases hosting genetic and other biological data, known as digital sequence information (DSI), are central to the implementation of the mechanism. This paper assesses the new international agreement and its implications for DSI databases. We walk through the database provisions in COP16 Decision 16/2, which include notifying users and submitters about the mechanism, improving metadata on geographical location of sample collection, and consistency with open access, as well as consideration of the FAIR, CARE, and TRUST principles. Drawing on surveys, interviews, and a workshop with biological database managers, we identify practical and scalable measures including updating terms of use, revising submission procedures, and strengthening user communication. We also propose approaches to capture and report non-monetary benefits such as capacity building, publications, interoperability, and training. These actions illustrate how DSI databases can remain open, sustainable, and globally connected while supporting benefit-sharing from the use of DSI on genetic resources.

## Introduction

Global biological databases provide open access to hundreds of millions of genetic sequences and many other data types and records derived from nature. These data are contributed daily by researchers worldwide and are essential for scientific research. The databases that underpin the life sciences enable the generation of knowledge that supports biodiversity protection, supports responses to pandemics and other global health crises, contributes to the mitigation of climate change, strengthens food security, and addresses many other challenges. These databases serve as essential sources of open reference data and cost database funders tens of millions of dollars annually to run^1^. For example, without a comprehensive and interoperable corpus of sequences, many genetic and taxonomic studies would be infeasible, as they depend on comparing new sequences against existing ones. This global corpus of data can also be leveraged using computational models and genomic tools to develop biotechnological innovations and commercial products and services. Accompanied by advances in synthetic biology, these data reduce the need for access to the biological material from which the sequences or other data were originally obtained.

For many countries that are Parties to the Convention on Biological Diversity (CBD) and its Nagoya Protocol^2^, open access to genetic data was perceived as a loophole in the bilateral Access and Benefit-Sharing (ABS) framework established by the Nagoya Protocol. While countries have sovereign rights to regulate (or not) access to their genetic resources and negotiate bilaterally whether and how benefits will return to their country, any use of public genetic data (and potentially other molecular biological data), widely referred to as Digital Sequence Information (DSI), was a grey area and thus benefits could not be guaranteed.

This tension emerged during international negotiations at the thirteenth CBD Conference of the Parties (COP13) in 2016^3^ and several years of debate and negotiation ensued. In 2022, at COP15 in Montreal, Canada, Parties agreed to establish a new multilateral mechanism (MLM) and a global fund for sharing benefits arising from the use of DSI that should be consistent with open access and should not hinder research and innovation^4^. In practice, maintaining open access to DSI meant that policy options such as paywalls and subscription models for accessing data from open databases were not compatible. Parties still have the option at the point of access to biodiversity to require that DSI derived from genetic resources cannot be made publicly available. In this case, researchers need to decide if they want to work in the country or not. However, national restrictions on DSI usage do not belong in the public DSI databases because that is not compatible with a multilateral handling of public DSI. These CBD design features safeguard the interconnectivity and interoperability of the life sciences database infrastructure and enable the reuse of data. The MLM therefore “decouples” access to DSI from the act of benefit-sharing, ensuring that open access is preserved, enabling research and innovation, while benefits are shared through a multilateral approach and a global fund.

At COP16 in 2024, in Cali, Colombia, Parties adopted Decision 16/2^5^ and operationalized the MLM by establishing the modalities to make financial contributions to the global fund launched in February 2025 (the “Cali Fund”)^6^. The decision also seeks the involvement of DSI databases in the implementation of the MLM. Although COP decisions are non-binding, they carry significant political weight, reflecting the consensus of the CBD’s 196 Parties^7^.

### What is the new DSI multilateral mechanism?

The MLM covers all publicly available DSI unless (future) benefit-sharing measures under other ABS instruments come into force^5,6^. The mechanism takes a sector-based approach for monetary contributions to the fund. Commercial entities that directly or indirectly benefit from the use of DSI, in sectors such as pharmaceuticals, cosmetics, agricultural, or biotechnology are expected to contribute 1% of their profits or 0.1% of their revenue to the Cali Fund if they exceed two of three financial thresholds: $20 million in assets, $50 million in sales or $5 million in profits^5,6^. With this, the MLM aims to provide new and crucial resources towards the US$200 billion spending gap for biodiversity conservation (Target 19 of the Global Biodiversity Framework)^7^.

A key outcome of COP16 for the global research community is the clarification that non-commercial entities such as public databases, public research and academic institutions, are not expected to provide financial contributions to the Cali Fund. However, the DSI decision does require all users of DSI, commercial and non-commercial, to share non-monetary benefits. The annex to decision 16/2 also indicates that both monetary and non-monetary benefits should be shared not only with Parties but also be directed toward the self-identified needs of Indigenous Peoples and Local Communities (IPLCs), who are often the custodians of the genetic resources from which the DSI originates.

### What does the new DSI multilateral mechanism require from DSI databases?

To support the operationalization of this mechanism, databases, tools and models that are dependent on DSI (hereinafter DSI databases) are requested to update their data governance policies to align with the MLM. Paragraph 10 of the Annex to COP16 Decision 16/2 sets out specific expectations for DSI databases^5^:

- DSI databases should inform users about the MLM and possible financial obligations arising from commercial use of DSI.
- DSI databases should inform submitters about: the need to comply with national and international legal obligations.
- DSI submitters should confirm that the DSI they submit to databases is not subject to any restrictions which prohibit its sharing.
- DSI metadata should include, where known, the location of collection of genetic resources and, where applicable, associated traditional knowledge.
- Databases should adhere to open access principles and consider the FAIR^8^, CARE^9^ and TRUST^10^ principles, as well as UNESCO’s Open Science Recommendation^11^.

In addition, Paragraphs 11 and 12, respectively, require CBD Parties and invite non-Parties (i.e., the USA) that fund, sponsor, or host DSI databases to take measures to ensure that DSI databases implement the COP16 decision^5^.

This paper aims to help DSI database managers support the CBD’s MLM. We outline simple, easily implementable changes to DSI database practices, and provide template language (see 10.5281/zenodo.20316423) to readily translate policy language into text that can be understood by scientists. Acknowledging that no single set of recommendations can fully reflect the global diversity of perspectives that could be relevant to the implementation of outcomes of Decision 16/2, we present these recommendations as a contribution to an ongoing dialogue. Our recommendations draw on 2.5 years of research, including a survey, expert interviews, workshops, and stakeholder working groups.

## What is a DSI Database?

At the time of writing, there is no globally agreed-upon definition of a “DSI database”, as the term *Digital Sequence Information* itself is not defined under the CBD or any other UN Fora. While DNA and RNA sequences are widely understood to be included within the DSI concept, it is still unclear whether DSI might also encompass protein sequences, epigenetic data, metabolites, macromolecular structures, or other digital representations associated with components of genetic resources.

Based on a study by Houssen *et al*.^12^ commissioned for the 2020 CBD Ad Hoc Technical Expert Group (AHTEG) on DSI, a four-tier classification for defining DSI was proposed:

- **Group 1 (Narrowest):** genetic information (DNA and RNA sequences).
- **Group 2 (Intermediate):** Group 1 plus amino acid sequences, molecular structures of proteins and epigenetic modifications.
- **Group 3 (Broader Intermediate):** Group 2 plus metabolites and other macromolecules.
- **Group 4 (Broadest):** Group 3 plus associated, contextual, and subsidiary information, such as associated traditional knowledge or ecological biotic and abiotic factors.

The AHTEG agreed that the first three groups could be considered DSI, while associated information (Group 4) is not^13^. Although this report has never formally been negotiated or agreed upon, we apply the Group 3 approach here for consistency, which captures the scale, diversity, and fluidity of the DSI ecosystem by encompassing the “informational content” of genetic resources.

The DSI definition, if ever agreed, would, in turn, determine what qualifies as a “DSI database”, a crucial consideration given the vast, diverse, and continuously evolving landscape of online resources that store, manage, analyze, and integrate biological data. Estimates for the number of biological data resources vary widely, depending on how they are counted, and range from 2,236 to nearly 7,000, with each managing tens of thousands to hundreds of millions of entries^14–16^. These resources range from general-purpose, comprehensive repositories to more specialized, domain-specific knowledge bases that together form an interconnected ecosystem^17^. Primary repositories such as the three databases of the International Nucleotide Sequence Database Collaboration (INSDC)^18^, and the Protein Data Bank^19^, aggregate and release sequence data, ensuring open access and submission-based access to primary data. Meanwhile, added value databases such as Ensembl^20^, UniProt^21^, SILVA^22^ and STRING^23^ build upon primary genomic, crystal structure, proteomic, and metabolomic data through functional annotations, integration of environmental metadata, connections between molecular pathways and phenotypic traits, and provide bioinformatic and taxonomic tools. All these resources cooperate with each other through data flows and interoperable formats^24^, allowing a genetic record to be interlinked to protein structures, metabolic pathways, and other integrated biological information and metadata.

There are also a vast range of DSI-related analytical tools, computational models, and high-throughput technologies, often available in specialized and generalist data and software repositories such as Galaxy^25^ and GitHub^26^. Bioinformatics platforms for sequence alignment and annotation, machine-learning models trained on extensive DSI datasets, and imaging technologies that produce high-resolution spatial gene expression maps generate large volumes of data in parallel to traditional DSI repositories^27^. These technologies, although not databases in the conventional sense, are increasingly relevant in both the generation and utilization of DSI.

Yet even with an operational definition of DSI based on the AHTEG report, many databases may be unclear whether they fall within the definition of a DSI database and, if so, whether they should implement the database requirements of the MLM.

One possible starting point for identifying key DSI databases might be the widely used 52 Global Core Biodata Resources listed by the Global Biodata Coalition. These resources are of fundamental importance to the wider biological and life sciences community and the long-term preservation of biological data^28,29^. However, even within this set, there are some resources which are clearly “DSI database”, such as the European Nucleotide Archive (ENA)^30^, and some which clearly are not, such as Europe PMC^31^, a literature database. There are some uncertainties as well. For instance, the Global Biodiversity Information Facility (GBIF)^32^ includes an increasing volume of DNA barcoding and metabarcoding records as vouchers for confirming species occurrence, but the majority of occurrence data within GBIF is derived from other evidence, such as specimen labels or human observations, and not from genetic data or other molecular data types. Similarly, resources such as Bgee^33^ and Reactome^34^ give extensive data on gene expression patterns and molecular pathways, respectively, but do not, and may not be able to provide direct links to the underlying nucleotide or amino acid sequences or any genetic diversity that underlies these expression data.

To support the implementation of Paragraph 10 of the Annex to Decision 16/2, further guidance from CBD Parties is needed. Clear and unambiguous criteria for determining which DSI databases, tools and models fall within the scope, combined with a structured process for dialogue, would support the effective participation of all platforms involved in the production, distribution, curation, and analysis of DSI, thereby advancing the practical operationalization of the MLM.

It is important to note that, given the wide variation in funding models and longevity, from publicly funded and permanent institutional repositories with many staff to privately maintained resources and project-based platforms run by just one or a few individuals, not all databases will have the capacity to fulfil all the expectations set out in Decision 16/2.

## How databases can support the MLM expectations in Paragraph 10

**Fig. 1:**
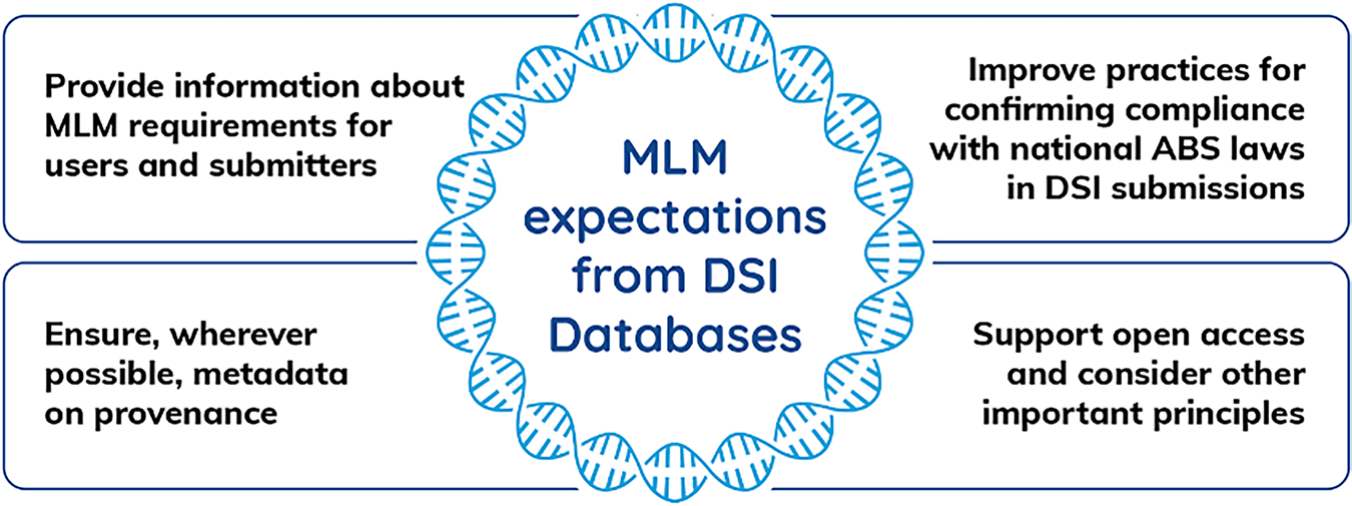
Expectations for DSI databases under the new CBD Multilateral Mechanism for benefit-sharing, as outlined in Paragraph 10 of the annex to Decision 16/2.

Despite the lack of a specific scope for DSI databases, many database managers view the expectations in Paragraph 10 of Decision 16/2 as aligned with established scientific best practices and ethical standards. This perspective was also reflected in a workshop held in March 2025 with database managers, where participants expressed a willingness to proactively engage with the MLM. Given the specialized nature of the database ecosystem, a phased, adaptive implementation led by the community of practice may be the most effective way to advance the recommendations set out below. This section outlines the key expectations for databases under the MLM, describes the status quo of database governance practices, and identifies steps to proactively support the implementation of the MLM. These are summarized in Fig. 1 and then described in detail.

### Provide information about MLM requirements for users and submitters

Paragraph 10 sub-paragraphs (a) and (b) call on databases to inform users and data submitters, respectively, about the MLM and possible financial obligations arising from commercial use of DSI, as well as the need to comply with national and international ABS obligations when accessing genetic resources.

#### Status quo

There are millions of DSI users worldwide and their activity is evident in the scale of data access. GenBank^35^ alone distributes about 1.3 terabytes each month and this volume is greatly exceeded by automated access through machine-to-machine interactions, such as large-scale downloads via Application Programming Interfaces (APIs) and File Transfer Protocol (FTP), which account for about 53 terabytes per month^36^. This high usage underscores the central role of reference sequence repositories in modern life sciences, where essential analyses, such as sequence comparison, annotation, and taxonomic assignment, depend on access to large, diverse, and geographically broad datasets.

**Fig. 2:**
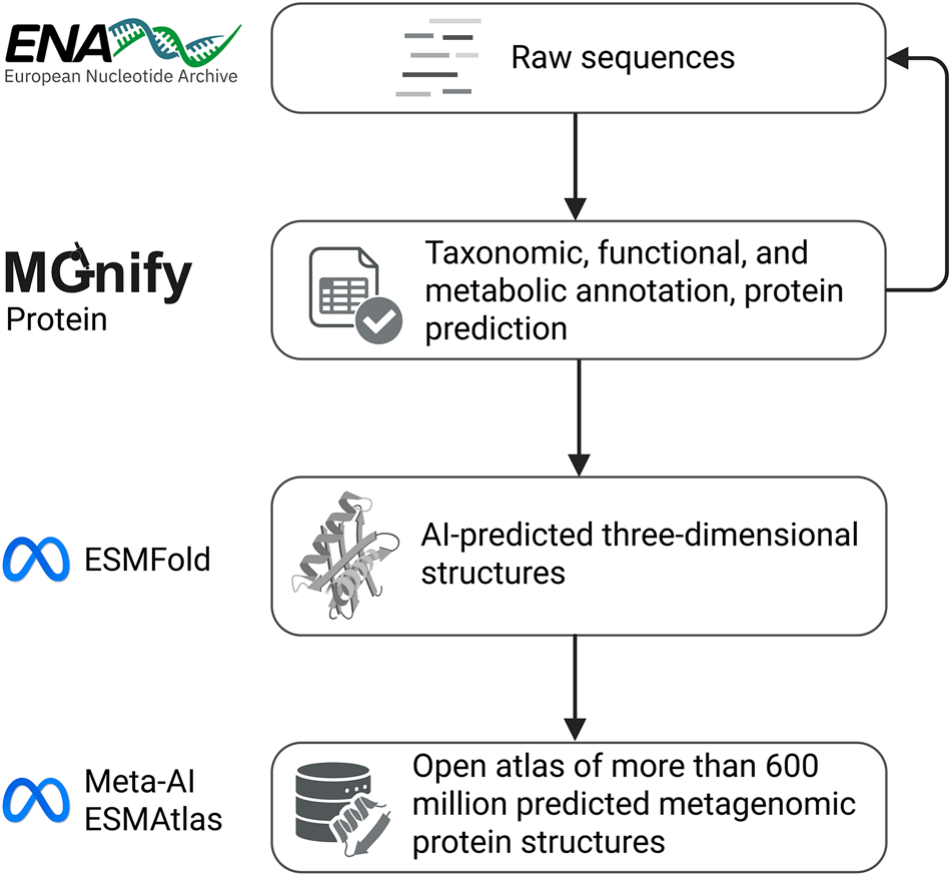
Complex data flows in DSI: The ENA–MGnify–ESMAtlas pipeline. Raw sequences deposited in ENA are processed by MGnify^83^, an automated pipeline that assesses the taxonomic, functional, and metabolic potential of environmental microbiomes. The processed data are integrated into MGnify Proteins^84^, which provides annotated protein cluster representatives derived from metagenomic assemblies. These annotated sequences are then used to build the ESM Metagenomic Atlas (ESMAtlas)^85^, a collection of more than 600 million AI-predicted metagenomic protein structures developed using ESMFold. In turn, ESMAtlas is currently included within FoldSeek^86^, a tool that enables large-scale comparison of protein structures. Furthermore, metagenome assemblies and analyses from MGnify, generated from ENA data, are linked back to ENA entries, making them accessible for reuse by the research community. This example illustrates how sequence data can move through multiple stages of processing and integration, ultimately reaching users in transformed and highly derived forms, far beyond the original repositories and sequences.

In addition, many interconnected databases, tools, and AI-based models act both as databases (and are therefore subject to Paragraph 10) and as regular users. They retrieve, process, and aggregate DSI, then redistribute it, driving its wide circulation across the global data ecosystem. To illustrate this, the chain of data resources involved in metagenomic protein structure prediction, as shown in Fig. 2, is instructive.

Because of these complex data flows that make use of the collectively available global repository of DSI, members of the scientific community recommended that DSI database governance focus on terms of use and global governance policies rather than attaching instructions to specific sequences^37^. Since policymakers largely followed this recommendation in decision 16/2, databases are now well-positioned to simply update relevant legal and policy information to users and submitters. These typically appear in Terms of Use or equivalent documents, which outline applicable legal obligations, data usage rights, data submitter responsibilities, and references to international standards.

#### Recommended Action

A practical and scalable step toward implementing sub-paragraphs 10(a) and 10(b) is revising the Terms of Use to include clear, accessible information about the MLM, its potential financial obligations, and instructing data submitters to comply with applicable national and international ABS regulations. To this end, we have proposed draft template language (10.5281/zenodo.20316423) which can be adapted and customized, as needed, to specific databases. The template language could help bridge the gap between policy language and scientific practice and support the thousands of databases that lack dedicated legal staff to navigate these issues.

Including links to guiding resources, such as the CBD’s informational page on the DSI MLM^38^, the Database Guide^39^ and the ABS Clearing House^40^ (where users can seek guidance on specific national obligations), would further enhance clarity and compliance. To reinforce the updated Terms of Use, databases could also consider communicating these changes through existing channels tailored to each database’s context, including social media, newsletters, or seminars. Additional implementation options could include pop-up notifications upon first access or at the time of data download requests, as well as news announcements.

### Improve practices for submitting DSI

Sub-paragraph 10(e) asks databases to request submitters of DSI confirm their submissions are not subject to restrictions that would prevent their sharing.

#### Status quo

Researchers must respect the terms and conditions established by the provider country regarding the production and sharing of DSI on genetic resources. Some countries include provisions in their ABS permits or mutually agreed terms (MAT) that restrict uploading DSI to open access databases. Therefore, users should check their permits before publishing sequence data to ensure that sharing is allowed and that they do not breach ABS agreements. Major DSI databases already have established procedures to support transparency and scientific integrity in DSI submission. The INSDC, for example, does not allow anonymous submissions. At ENA, submitters are required to agree at the time of registration that the DSI they submit are not sensitive or restricted-access. This agreement is confirmed through a checkbox as part of the account creation process. Although submitters are currently not asked to reconfirm these terms for each individual submission, it is understood that continued use of the system implies ongoing acceptance of the database’s terms and conditions.

#### Recommended Action

Databases could ask submitters to actively confirm, for each dataset uploaded or bulk submissions, that the DSI is not subject to legal restrictions on sharing. This could be implemented through a checkbox at the point of submission, consistent with existing user workflows.

The effectiveness of this measure will depend on the clarity and appropriateness of the language used. Language that is too complex or unclear could discourage data submission, ultimately weakening the value of public DSI repositories, which fundamentally rely on the volume of the DSI corpus for reliable and statistically sound query results. On the other hand, if the language is too vague or simplistic, submitters may confirm compliance without fully understanding their responsibilities, potentially undermining the credibility of the mechanism. Here again, the proposed template language (10.5281/zenodo.20316423) could support submission agreements which could be accompanied by references to relevant legal frameworks and guidance materials.

Such resources would also be valuable for scientists who wish to create new databases from scratch, including cases where database curators act as data uploaders or submitters. In these contexts, secondary databases may reasonably rely on primary databases having obtained confirmation from original submitters. However, in case they accept direct submissions from researchers or integrate data which were not yet made publicly available on DSI databases, they should apply the full package of measures to ensure compliance.

### Ensure, wherever possible, metadata on provenance

Sub-paragraph 10(c) asks databases to “require the provision of information on the country of origin of the genetic resources from which the DSI was derived, where known, as well as, when appropriate, additional metadata”, including associated traditional knowledge with those genetic resources and their origin or source.

#### Status Quo

**Fig. 3:**
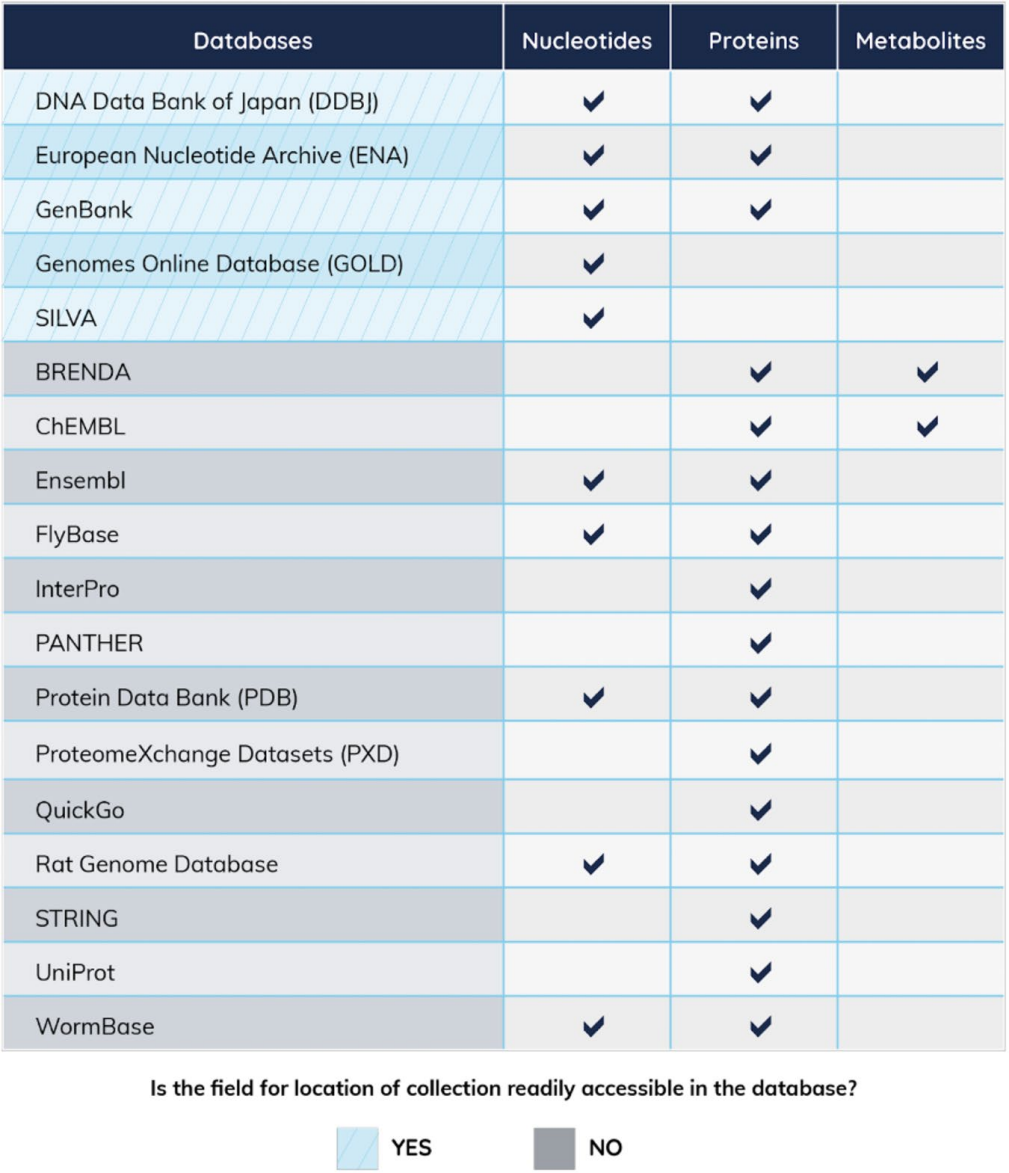
Analysis of 18 key biological databases considered in scope of the CBD (i.e., databases which contain non-human data and data collected from the wild). Databases are categorized based on the types of DSI they host (nucleotide sequences, protein sequences, and metabolite data) and whether country-of-origin metadata were present in the spot-checked datasets. Note: Country of origin refers to the geographical location of collection of the original genetic resource from which the DSI was derived. (Figure adapted from Raposo *et al*. 2023^87^).

Reporting of country of origin information (which refers to the geographical location of collection of the original genetic resource from which the DSI was derived) in DSI databases shows a high degree of variability across primary and downstream resources. As part of a preliminary assessment in 2023, we carried out a spot check of selected biological databases, including a set of those on the list of Global Core Biodata Resources^28^ and additional community-maintained databases in Germany, to examine the availability of metadata on location of collection (see Fig. 3, and methods section). Of the 43 databases examined, 18 were considered within the CBD scope (i.e., databases which contain non-human data and data collected from the wild). Among these 18, only five included a visible metadata field for location of collection (Fig. 3 and methods section). These five databases either focus exclusively on nucleotide sequences (DNA/RNA) or are part of the INSDC: DDBJ (DNA Data Bank of Japan)^41^, ENA, and GenBank. In contrast, the remaining 13 databases, which primarily aggregate protein sequences, metabolites, or biological pathways, did not provide a readily detectable field for the collection location.

However, the existence of a metadata field for the location of collection does not guarantee that this information is given. Although INSDC has provided a dedicated field for this purpose since 1998, as of 2020, only 16% of the sequence entries included a country tag^36^. The INSDC introduced a mandatory requirement in March 2023 for submitters to provide provenance information (aka spatio-temporal information, i.e., location of collection and date of sampling) for all new environmentally-sourced sequence entries. Recent data indicate that the new requirement, together with increased awareness of the FAIR principles^8^, has significantly improved provenance metadata reporting. In the past five years, INSDC has received 56.9 million sequences, of which 25.9 million (46%) include information on the geographical location of collection, a substantial increase compared with the entries before 2020. Nevertheless, when considering all available sequences, only 51.9 million out of 307 million (17%) include a geographical location of collection.

Several factors contribute to this gap in information. First, human data, which accounts for 12% of the dataset^36^, is often not suited to have provenance information because of privacy concerns. Second, provenance information on the geographical location of collection was and remains not feasible for DSI from model organisms. These organisms comprise around a third of the global DNA dataset^36^, where the original collection location was unknown, as well as for cosmopolitan species found everywhere, where collection location was not prioritized. Furthermore, curated or downstream databases face scientific and technical challenges in attributing data to a specific location of collection if the sequence is found in many different organisms or across the tree of life. These databases aggregate knowledge from diverse sources, making it difficult or impossible to assign a single provenance. For instance, pathway databases such as MetaCyc^42^ describe the ubiquitous Krebs cycle, also known as the tricarboxylic acid (TCA) cycle, integrating decades of research across species from microorganisms to humans. In such cases, assigning a specific location of collection to a scientific database presenting a biologically universal process would be neither accurate nor meaningful from a scientific perspective.

From the perspective of database managers, a significant challenge lies in tracing the propagation of DSI and its associated metadata, especially for databases that host aggregated data. Currently, there are no clear, standardized mechanisms to ensure consistent and accurate metadata transfer across the complex chains of data processing and integration. Metadata are the result of an incremental and continuous enrichment and merging process^43^ throughout the research data lifecycle^44^. This begins with experimental planning and data collection, through data preparation, analysis and processing, and ends with publication in databases as the final step, followed by reuse in subsequent cycles. Metadata may appear in unstructured form (e.g., electronic laboratory notebooks) or structured form (e.g., internal databases across different responsibility areas)^45^. Clear guidelines are therefore essential to outline the metadata to be collected for each phase.

One example of best practice in annotating biological resources is the comprehensive yet selective description of plant genetic materials in the BioSamples database^46^ (https://www.ebi.ac.uk/biosamples/samples/SAMEA104630643). Ideally, samples should include essential metadata, such as geotags and precise collection locations, to ensure all relevant information is linked and easily accessible. Annotations should be limited to a small set of relatively stable information and then linked to well-established aggregator information systems in the field of plant genetic resources such as the Global Information System for Plant Genetic Resources for Food and Agriculture (GLIS, https://glis.fao.org/glis/). Multi-Crop Passport Descriptors (MCPD) should not be copied and pasted into BioSamples since it is continuously curated by genebanks and would quickly result in outdated entries. Additionally, when referencing materials registered in databases like GLIS, users should always cite them using their Digital Object Identifiers (DOIs) whenever available^47^.

#### Recommended Action

All DSI databases, wherever possible, should follow the lead of the INSDC by requiring submitters to provide provenance information, i.e., the location and date of collection of the genetic resource from which the DSI was derived, at the point of submission, with a particular focus on environmentally-derived data. This not only supports the MLM but is consistent with good scientific practices. If not already available, dedicated metadata fields (location and date) should be introduced to collect this information, ideally in a similar manner to the INSDC. On top of that, databases should expand their metadata schemas to include structured fields that allow submitters to indicate whether the DSI has traditional knowledge associated with the genetic resource originally collected, its origin or source.

Given the vast number of existing records, with over 4.76 billion assembled sequences in INSDC alone^18^, it is neither feasible nor scientifically appropriate to retroactively add missing provenance information. Efforts should instead focus on improving the quality of metadata for new submissions. Where possible, databases should encourage contributors to provide more detailed geographic information, such as latitude and longitude coordinates based on a standardized system of coordinates (e.g., World Geodetic System 1984 - WGS84^48^), as higher spatial resolution in sequence metadata brings clear benefits. However, more precise geographic data must be managed responsibly. For example, guidelines for generalizing the locations of sensitive or endangered species should be applied to balance the value of detailed metadata with the need to protect vulnerable biodiversity.

The issue of how metadata should be inherited, transformed, or augmented when DSI is used to create new datasets or analyses remains a complex challenge and will also challenge other ABS instruments such as the new High Seas Treaty, officially known as the Agreement under the UN Convention on the Law of the Sea on the Conservation and Sustainable Use of Marine Biological Diversity of Areas beyond National Jurisdiction (BBNJ Agreement)^49^, the International Treaty on Plant Genetic Resources for Food and Agriculture (ITPGRFA)^50^, and the World Health Organization Pathogen Access and Benefit-Sharing System (WHO PABS)^51^. Efforts toward metadata standardization and harmonization would support transparency and interoperability across databases and promote integration of activities across different ABS instruments but, as discussed above, it will not always be possible or scientifically appropriate to proliferate provenance information where DSI is merged and/or highly conserved across the tree of life.

### Support open access and consider other important principles

Decision 16/2 acknowledges the essential role of open access to DSI in the preamble and, in sub-paragraph 10(d) of the annex, calls for databases to be consistent with open access practices while taking into consideration the FAIR, CARE and TRUST principles, as well as the UNESCO Recommendations on Open Science.

#### Status quo

Major biological sequence databases operate on a strong foundation of open access, a practice that traces back to the Bermuda Principles (1996)^52^, the Fort Lauderdale Agreement (2003)^53^, and the Toronto Agreement (2009)^54^. These agreements established rapid public release and unrestricted use of DNA sequence data as a standard in genomic research to enhance scientific collaboration and public benefit and maximize impact of public funding. This openness enables the DSI ecosystem to function at scale, supporting millions of daily queries, powering downstream use across interconnected databases, tools, and models, and ensuring that researchers worldwide, including in Low- and Middle-Income Countries, can access and use DSI without technical or financial barriers. However, despite the wide recognition of the importance of open access, interpretations of what it means can differ in practice: the 100 + members of the DSI Scientific Network^55^ see scientific value in the INSDC model (anonymous, free of charge, reusable, and machine-readable)^54,56^, whereas recently some have suggested that database managers could and, indeed should, police their users and inform supranational entities about who has access data, thus fundamentally altering current practice^57^. A shared definition of open access is therefore crucial for consistent implementation.

Maintaining an open access infrastructure is costly and requires skilled personnel^1,36^. Indeed, many biological databases are discontinued each year due to limited resources (or lack of demand)^14,58^ potentially upwards of 40%^59^. In light of these challenges, the explicit recognition in the CBD Decisions (15/9 and 16/2)^4,5^ of the importance of open access infrastructure offers an opportunity to mobilize broader stakeholder support and promote sustainable investment, such as the efforts by the Global Biodata Coalition^60^. This is essential not only for the long-term viability of databases and related services but also for addressing DSI capacity gaps, including the uneven global capacity to generate sequence data and to fully benefit from the open-access services provided by these databases. While most major data infrastructures remain concentrated in high-income countries^16^, important national and regional initiatives are emerging to build sequencing and data infrastructure in other parts of the world. Notable examples include the African Bioinformatics Institute (ABI)^61^, African BioGenome Project (AfricaBP)^62^, the Genomics of the Brazilian Biodiversity (GBB) initiative^63^, and the Hong Kong Earth BioGenome Project (EBP-HK)^64^. To prevent the emergence of regional silos, it is important that these initiatives operate in alignment and interoperability with the INSDC. To that end, the INSDC Global Participation Initiative^18,65^, is an essential next step in the DSI globalization trend.

Decision 16/2 also noted the FAIR, CARE, and TRUST principles and the UNESCO Recommendation on Open Science^5^. Over the past decade, biological sequence databases have increasingly aligned with the FAIR principles, first introduced in 2016^8^, which call for data to be Findable, Accessible, Interoperable, and Reusable. Today, these principles are widely implemented across major infrastructures. The TRUST principles, introduced in 2020^10^, complement FAIR by offering a framework to enhance the long-term trustworthiness of data repositories. They emphasize transparency, responsibility, user focus, sustainability, and appropriate technology as the core elements for ensuring that data infrastructure remains robust, governed responsibly, and adaptable to evolving technology and stakeholders’ requirements.

The CARE principles, also published in 2020^9^, emphasize Collective benefit, Authority to control, Responsibility, and Ethics in the context of Indigenous data governance. While DSI databases seek to support these principles where possible, they also highlight practical challenges, particularly with the principle of “Authority to Control”. As there is no central authority to manage or enforce access permissions for Indigenous data, this requirement becomes difficult to implement across many mainstream DSI databases and raises potential liability concerns and incompatibility with open access.

#### Recommended action

Future changes to data governance under the MLM must safeguard open access (anonymous, free of charge, reusable, and machine-readable), a principle for scientific progress and equitable benefit-sharing^37^. At the same time, biological databases should aim to engage with the CARE principles where feasible^37^. For example, if DSI obtained or shared unethically is identified, databases should take appropriate steps to remove or retract the affected records.

More broadly, several practical measures can help align database practices with the CARE principles. One is promoting transparency around data provenance. This can include, when applicable, displaying relevant metadata linked to the genetic resource that generated the DSI, such as collection permits or Biocultural Labels^66^ that reflect community expectations for appropriate use of data. Databases can also adopt more inclusive governance structures by involving representatives from Indigenous Peoples and local communities in advisory boards, steering committees, or data access discussions. To support such implementations, operational guidance tailored to biological data repositories would be highly beneficial. Developing frameworks, such as existing ones developed for the domain of Earth sciences^67^, for the DSI context would support more consistent and principled data governance across the global database landscape. The GBIF indigenous data governance task group^68^ provides a platform for exploring the compatibility of fully open biodiversity data platforms with the application of the CARE principles, including DSI.

## How databases can support the MLM in making non-monetary benefits from DSI visible

Beyond the expectations outlined in paragraph 10, DSI databases are uniquely positioned to generate and measure non-monetary benefits as required under the CBD DSI Decision 16/2. Unlike commercial users of DSI, who typically do not deposit their sequences and data in public, open access databases, researchers and databases contribute open access DSI, open datasets, and scientific publications. All these contributions support biodiversity conservation, sustainable use, and the achievement of the Kunming-Montreal Global Biodiversity Framework (KMGBF)^7,69^. Non-monetary benefits can also include a wide range of activities such as capacity building, collaborations in research and/or product development, training programs and technology transfer to strengthen local expertise and infrastructure. Although non-monetary benefits do not involve direct financial transfers, and are not a replacement for monetary benefits^5^, these contributions represent a significant financial investment.

Non-monetary benefits from DSI need to be quantified and made visible globally to contribute to increasing benefit-sharing from the use of DSI in line with Goal C and Target 13 of the KMGBF.

The headline indicator C.2 of the KMGBF Monitoring Framework, calls for measuring “non-monetary benefits arising from applicable international ABS instruments”, which will be reported and aggregated in a comparable way. In particular, a new global methodology^70^ was developed to measure three types of non-monetary benefits from scientific publications: research and development results, joint scientific publications, and scientific publications relevant to priority areas such as conservation, sustainable use, public health and food security. This methodology could be further developed and applied to DSI. For example, the number of publications that cite DSI could be assessed as a proxy for the number of research results arising from the use of DSI. These scientific publications could also be classified in the mentioned four priority research areas^70^. Some databases are already evaluating ways to track citation of DSI records in scientific publications^71^. Measuring these indicators would increase visibility of research results and scientific publications as non-monetary benefits from the use of DSI. Care will need to be given to ensure that these measurements also consider the quality or social impacts of such data.

Beyond the KMGBF headline indicator C.2, the MLM should recognize open access to DSI as a type of non-monetary benefit. Generating sequences and hosting them in open databases costs money and represents a significant investment. This aligns with other ABS instruments, such as the BBNJ Agreement, and underscores its value in generating reliable data for biodiversity research and conservation. The MLM could also collect information related to DSI database content, such as the number of DSI entries available in open access databases. This information could also be disaggregated by country or location of collection of the genetic resource from which DSI was generated, contributing to identifying gaps in DSI production and guiding capacity building efforts^72^. In addition, when available, sample-level metrics can serve as a valuable complementary proxy, as a single biological sample often generates many individual DSI records, as in the case of BioSamples^46^. Linking DSI to biological samples could be further supported by emerging approaches such as large language models for metadata integration^73^. Apart from data availability, the volume of data exchange and interoperability between DSI databases can also be measured by monitoring the number of interlinked DSI databases and API integrations. These non-monetary benefits reflect how well the DSI ecosystem functions as a network^24^ in order to provide valuable and usable DSI for the scientific community.

DSI databases also have anonymized information on their users and could share an estimated number of users of public DSI databases, disaggregated by country, based on anonymized IP data. While not without limitations, usage statistics offer insights into DSI access patterns, demonstrating open access benefits for global researchers, including those in low- and middle-income countries, and revealing capacity gaps in using DSI. Similarly, mapping the geographic location of databases and their primary funders, particularly those that have taken steps to comply with Paragraph 10, can illustrate global DSI infrastructure patterns and capacity gaps, and identify alignment efforts with the MLM. This mapping could also help generate infrastructure-related indicators that serve as proxies for non-monetary benefits. Finally, DSI databases often educate their users on how to use the data resources^74^. Here, measuring the number of capacity-building efforts provided by databases, such as courses and learning materials, would collectively highlight how databases contribute to building scientific capacity to use DSI.

These non-monetary benefit-sharing indicators should not impose new burdens on database managers but rather aim to highlight databases’ existing contributions to the DSI ecosystem and foster feasible voluntary actions within their current staffing and financial capacities. The indicators would only be feasible if supported by third parties. For example, funding could come from entities like the Cali Fund to cover the effort involved in collecting these metrics. The metrics could be structured as core and optional indicators, with core indicators establishing the minimum data-sharing requirements for participating databases. Centralized platforms or automated aggregation systems, including a CBD-led Clearing House, could support data integration and minimize the operational burden on individual databases.

Furthermore, ensuring long-term and continuous support for these data-sharing and database participation structures will be essential. A phased implementation approach, including possible pilot phases to monitor functionality, could help achieve this. Additionally, integration with existing infrastructures, such as GBIF, and support from the CBD and its subsidiary bodies could further facilitate the implementation process.

The system of data citation developed by GBIF with the use of digital object identifiers (DOIs) could provide a model for cost-effective monitoring and communication of downstream data use^75–77^.

Such metrics would also encourage coordination between the MLM and other international benefit-sharing instruments (e.g., ITPGRFA, WHO PABS, BBNJ Agreement). By contributing indicators that potentially support multiple monitoring frameworks, DSI databases can help encourage alignment across the international instruments and reduce fragmentation in the implementation of their metrics. This approach reflects the practical reality that DSI is integrated and “mixed together” into a global dataset, irrespective of the specific UN forum in which it is discussed^78^.

## Future perspectives

The success of future data sharing and management will depend not only on their technical robustness and reliability, but also on their ability to accommodate existing cultural and legal changes. In this context, we highlight three pressing areas that will shape the future trajectory of biological data management and their governance: the standardization of terminology, the role and limitations of general-purpose repositories, and the emergence of new data modalities such as imaging.

### Standardization of terminology

One of the key barriers to effective data sharing is the inconsistent use of terminology across jurisdictions. A prominent example is the term “country of origin”. Under the CBD framework, this term often carries legal weight, linking biological samples to questions of data sovereignty and benefit-sharing. However, its interpretation varies widely: some countries consider ancestral provenance, while others, including much of the scientific community, refer to the location *in situ* where the data were acquired. Similar inconsistencies arise with terms such as “data owner” or “data provider.” Scientists typically use these terms to indicate the person responsible for generating and/or uploading the data, whereas in policy discussions, they can be interpreted as referring to the provider country of the genetic resource, again implying sovereignty interpretations. This heterogeneity in terminology has caused confusion and, at times, friction in data management policies.

The global research community must engage in building consensus around the semantics of “origin” and “provider” in biological data. Without such consensus, ambiguities will continue to complicate both scientific interpretation and international negotiations under the CBD. Establishing common ground will likely require multi-stakeholder dialogue involving database managers, researchers, policymakers, legal experts, and representatives from indigenous and local communities. The goal should be to balance the legitimate rights and interests of data contributors with the need for interoperability and clarity in scientific data standards.

### The role and limitations of general-purpose repositories

In parallel with the expansion of domain-specific databases, there has been a proliferation of general-purpose repositories such as Zenodo^79^, Figshare^80^, and others. These repositories have played a pivotal role in promoting open science, particularly by lowering the barriers for researchers to deposit and disseminate data regardless of its type, format, or disciplinary background. They have also been embraced by funding agencies and journals as a convenient vehicle for ensuring data availability in compliance with open access mandates.

However, the very strengths of general-purpose repositories also present limitations. Because these platforms can accommodate diverse forms of data, from astrophysical simulations to social science survey results, they often require only minimal metadata at the point of deposition. This “lightweight” approach is attractive for rapid dissemination but undermines the long-term usability of the data. Without standardized metadata, integrating such datasets into broader infrastructures or reusing them across disciplines becomes difficult, if not impossible.

Thus, while general-purpose repositories will remain an important component of the open science ecosystem, they cannot substitute for domain-specific, curated repositories when it comes to ensuring long-term sustainability, interoperability, and compliance. A critical task for the coming decade will be to establish mechanisms for bridging the gap between general-purpose and domain-specific repositories or clearing houses. This may involve AI-based metadata enrichment pipelines, stronger community curation practices, or clearer guidance from funding bodies and journals on which repositories are appropriate for different data categories.

### The emergence of new data modalities

A third area of growing importance concerns the rise of new data modalities, particularly in the realm of imaging. Advances in imaging technologies, from ultra-high-resolution microscopy to image-based gene expression, are producing vast volumes of data that are both scientifically valuable and challenging to manage^81^. Unlike traditional genetic data, images often carry dual identities: they are simultaneously scientific records and creative works potentially subject to copyright law.

There is currently no international consensus on how to treat these emerging data types within the framework of CBD governance. Do they fall under existing copyright regimes, or should new categories be created to address their unique characteristics? How should issues of consent and benefit-sharing be handled when images include culturally or ecologically sensitive sites?

Addressing these questions will again require international dialogue and the development of shared principles. Lessons may be drawn from the evolution of data-sharing norms in genomics, where early concerns about misuse and misappropriation gave way to structured agreements and metadata standards that facilitated both openness and accountability^10,52,53^. Similar tracks may be needed for imaging data, involving recognition of their dual scientific/legal identities, and the creation of metadata standards tailored to images.

## Conclusion

With clear guidance, collaborative engagement between database managers, users, submitters, and countries, DSI databases can help operationalize the MLM transparently, efficiently, and inclusively. Upholding the expectations outlined in Paragraph 10 is not only a matter of policy compliance but also part of good scientific practice and adherence to research integrity and ethical standards. Leading DSI databases, such as the INSDC^18^ and key Global Core Biodata Resources (GCBRs)^29^ should take the lead in implementing these measures, setting a practical and scalable example for smaller, less-resourced, and secondary databases. The Global Biodata Coalition could also play an important role by facilitating the engagement of additional GCBRs as the process develops. By engaging proactively, database providers can help ensure that future CBD and other policy decisions are grounded in the realities of scientific work. Early, coordinated involvement is essential to preserving the openness and functionality of the current DSI data infrastructure. In this context, supporting the MLM is closely aligned with efforts to advance robust, open, and sustainable data systems taking care not to attribute a policing role to databases for MLM compliance. Enhancements such as improved metadata, clearer terms of use, as well as better user communication and awareness-raising directly promote scientific transparency, reproducibility, and data reusability. These actions fortify the integrity and global utility of DSI repositories, thereby reinforcing their value to funders, researchers, and policymakers.

## Methods

We manually assessed DSI databases for the availability of metadata that could support the development of the multilateral benefit-sharing mechanism on Digital Sequence Information (DSI) and the Cali Fund. Our analysis focused on two main sources: databases identified as Global Core Biodata Resources (GCBRs) by the Global Biodata Coalition^29^, and selected community-led databases maintained by members of the German National Research Data Infrastructure (NFDI)^82^. The GCBRs are recognized as essential resources for the global biological and life sciences community, playing a crucial role in the long-term preservation and accessibility of databases. The NFDI databases play a similar role nationally within Germany.

For each database, we first assessed whether its scope aligned with the Convention on Biological Diversity (CBD). Databases exclusively handling data on model organisms (i.e., species maintained in laboratory settings prior to 1993) and/or human genetic data were considered outside the CBD scope and excluded from further analysis.

In total, we analyzed 43 databases. 18 databases were deemed likely to be within CBD scope. We then randomly examined datasets in the databases to check the availability of a metadata field for the country of origin of the genetic resource from which the DSI was obtained.

In addition, we categorized databases based on the types of biological information they host, following the groups approach proposed by the study for the Ad Hoc Technical Expert Group (AHTEG) on DSI in 2020:

- DNA and RNA sequences,
- Protein sequences or structural data and/or epigenetic modifications,
- Metabolites and macromolecules.

The results of this analysis are summarized in Fig. 3.

## Data Availability

The results of the metadata analysis described in the Methods section are available in .xlsx format on Zenodo: 10.5281/zenodo.20083956.

## Code availability

No custom code was generated or used.
